# Rapid Detection of Imipenem Resistance in Gram-Negative Bacteria Using Tabletop Scanning Electron Microscopy: A Preliminary Evaluation

**DOI:** 10.3389/fmicb.2021.658322

**Published:** 2021-06-16

**Authors:** Gabriel Haddad, Anthony Fontanini, Sara Bellali, Tatsuki Takakura, Yusuke Ominami, Akiko Hisada, Linda Hadjadj, Jean-Marc Rolain, Didier Raoult, Jacques Yaacoub Bou Khalil

**Affiliations:** ^1^Institut Hospitalo-Universitaire Méditerranée Infection, Marseille, France; ^2^Aix-Marseille Université, Institut de Recherche pour le Développement (IRD), UMR Microbes Evolution Phylogeny and Infections (MEPHI), Marseille, France; ^3^Hitachi High-Tech Corporation, Analytical & Medical Solution Business Group, Ibaraki, Japan; ^4^Hitachi High-Tech Corporation, Nanotechnology Solutions Business Group, Toranomon Hills Business Tower, Tokyo, Japan; ^5^Hitachi, Ltd., Research & Development Group, Tokyo, Japan

**Keywords:** gram-negative bacilli, imipenem, microbiology, rapid AST, scanning electron microscopy

## Abstract

**Background:** Enabling faster Antimicrobial Susceptibility Testing (AST) is critical, especially to detect antibiotic resistance, to provide rapid and appropriate therapy and to improve clinical outcomes. Although several standard and automated culture-based methods are available and widely used, these techniques take between 18 and 24 h to provide robust results. Faster techniques are needed to reduce the delay between test and results.

**Methods:** Here we present a high throughput AST method using a new generation of tabletop scanning electron microscope, to evaluate bacterial ultra-structural modifications associated with susceptibilities to imipenem as a proof of concept. A total of 71 reference and clinical strains of Gram-negative bacteria were used to evaluate susceptibility toward imipenem after 30, 60, and 90 min of incubation. The length, width and electron density of bacteria were measured and compared between imipenem susceptible and resistant strains.

**Results:** We correlated the presence of these morphological changes to the bacterial susceptibility and their absence to the bacterial resistance (e.g., *Pseudomonas aeruginosa* length without [2.24 ± 0.61 μm] and with [2.50 ± 0.68 μm] imipenem after 30 min [*p* = *3.032E-15*]; *Escherichia coli* width without [0.92 ± 0.07 μm] and with [1.28 ± 0.19 μm] imipenem after 60 min [*p* = *1.242E-103*]). We validated our method by a blind test on a series of 58 clinical isolates where all strains were correctly classified as susceptible or resistant toward imipenem.

**Conclusion:** This method could be a potential tool for rapidly identifying carbapenem-resistance in *Enterobacterales* in clinical microbiology laboratories in <2 h, allowing the empirical treatment of patients to be rapidly adjusted.

## Introduction

Rapid antibiotic susceptibility tests (ASTs) has been an important topic in clinical microbiology in recent decades (Jorgensen and Ferraro, 2009; Syal, 2017; van Belkum et al., 2019). Rapid AST helps to speed up diagnosis, leading to targeted antibiotic therapy as early as possible (Wolk and Johnson, 2019). Moreover, it is now well-established that early administration of appropriate antibiotics is the key factor associated with clinical outcome in patients with sepsis (Levy et al., 2018). Multiple systems have been described for the early detection of antibiotic resistance in bacteria. Methods using molecular biology and genetics can deliver AST results within 3 h but only enable the detection of known antibiotic resistance genes (Ellington et al., 2017; She and Bender, 2018). Early detection by culture-based methods coupled with optical microscopy, video imaging, micro-fluidic approaches, immunoassays, bio-sensors, and machine learning (Mohan et al., 2013; Kelley, 2017; Shi et al., 2018), yielding results within 3 to 6 h (Le Page et al., 2015; Choi et al., 2017; Smith et al., 2017), have been reported and are proposed as a possible way to replace routinely used methods. Other common AST methods used in clinical laboratories are broth micro-dilution, disk diffusion and E-test methods (Baker et al., 1991). Although high speed methods are efficient in profiling microorganisms for susceptibility or resistance, many barriers still prevent their global usage or adaptation as gold standard methods.

Bacterial ultra-structural modifications after antibiotic contact have been previously described using optical, scanning, and transmission electron microscopes (Greenwood and O'Grady, 1969; Iida and Koike, 1974; Choi et al., 2017; Zahir et al., 2019), but were not used so far in the clinical context due to the extended sample preparation methods, high cost and expertise required. Here, we demonstrate the use of a new generation of rapid tabletop scanning electron microscopes (SEM) to assess the bacterial morphology after incubation with imipenem, by analyzing the bacterial ultra-structure and their morphological modifications instead of their growth using SEM. We targeted seven of the most common Gram-negative bacterial species isolated from clinical samples in order to identify potential ultra-structural modifications that can be used to discriminate between resistant and susceptible bacteria when incubated with imipenem (IPM) and associated these changes with a resistance or a susceptibility profile to this key antibiotic. We also tested a *Stenotrophomonas maltophilia* isolate, a Gram-negative bacilli known to be naturally resistant to IPM (Howe et al., 1997). The first tests were carried out on reference strains with known susceptibility profiles toward IPM. We then applied our newly developed method blindly to a series of 58 clinical isolates with different profiles toward IPM to validate our technique.

## Methods

### Ethics Statement

The studies involving human participants were reviewed and approved by the ethical committee of the University Hospital Institute Méditerranée Infection (19-21 Boulevard Jean Moulin, Marseille, France).

### Sample Collection

Seven of the most common Gram-negative bacilli species were selected for this study. We used *Pseudomonas aeruginosa* (CSURP9558, CSURP9559)*, Klebsiella pneumoniae* (CSURP9552, CSURP9553)*, Escherichia coli* (CSURP9546, CSURP9547)*, Enterobacter cloacae* (CSURP9548, CSURP9549)*, Proteus vulgaris* (CSURP9556, CSURP9557)*, Proteus mirabilis* (CSURP5544, CSURP8541)*, Acinetobacter baumannii* (CSURP9540, CSURP9541), and *Stenotrophomonas maltophilia* (CSURP5256) reference isolates susceptible and resistant toward IPM collected from the “*Collection de Souches de l'Unité des Rickettsies*” (*CSUR*, WDCM 875).

For each of species, clinical isolates were also collected from the Institut Hospitalo-Universitaire Méditerranée Infection microbiology laboratory isolated between the years 2017 and 2019 (Supplementary Table 1). Colonies were then collected and re-suspended in Protect Microorganism Preservation System media (Technical Service Consultants Ltd, Lancashire, UK) and conserved at −80°C for further analysis.

### Sample Profiling

**Figure 1:**
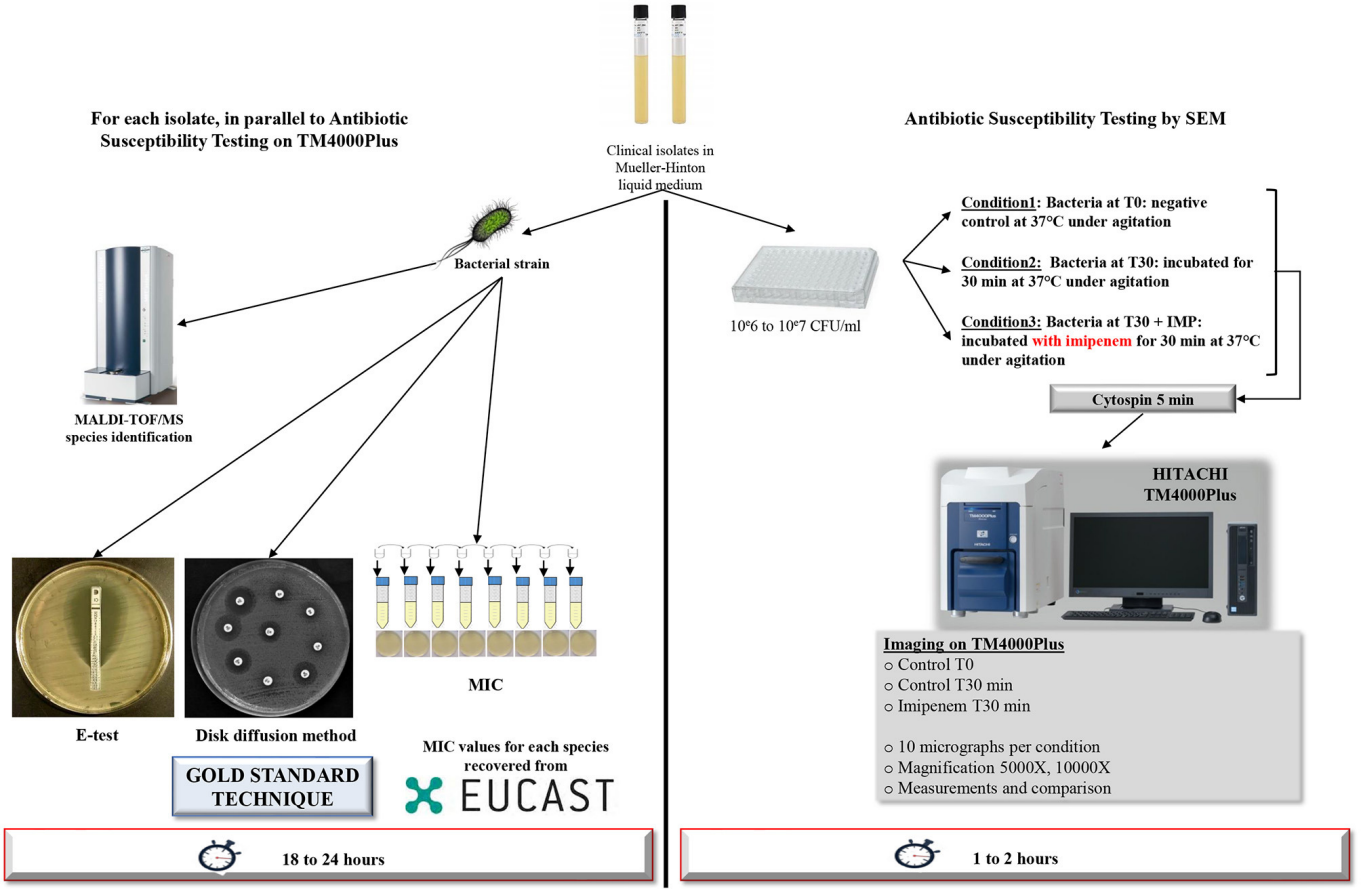
Workflow of the strategy used. Detailed strategy applied to a selection of Gram-negative bacilli for resistance toward IPM tests after 30 min of incubation using TM4000 Plus tabletop scanning electron microscope. For other time-points, the same strategy was applied.

All isolates were checked by MALDI-TOF/MS (matrix-assisted laser desorption/ionization time-of-flight mass spectrometry; Microflex, Bruker Daltonics, Germany) for identification prior to each experiment (Seng et al., 2009). We also checked the antibiotic susceptibility for each isolate and determined the MICs using the E-test technique (BioMérieux), broth micro-dilution, and disk diffusion method were performed as the gold standard AST procedures for IPM resistance profiling. Incubation took place at 37°C for 18 to 24 h. The whole process is shown in Figure 1. We then carried out an RT-PCR assay on IPM-resistant strains, targeting six β-lactamase resistance genes (blaNDM-1, blaOXA-48, blaOXA-23, blaOXA-58, blaKPC-2, blaVIM) as well as the OprD truncated gene for *P. aeruginosa*, as described (Diene and Rolain, 2014). All PCR were performed in duplicate.

### Antimicrobial Susceptibility Testing Assay Using a Tabletop Scanning Electron Microscope TM4000 Plus

#### Sample Preparation

Bacterial colonies were cultured in liquid Mueller-Hinton media (MH) and quantified using McFarland standards to adjust the viable bacteria to 10^6^ to 10^7^ CFU/mL for AST. Bacterial suspensions were then cultured with IPM at the breakpoint MIC of EUCAST (Supplementary Table 1) and incubated at 37°C under agitation, for a selected time point ranging from 30 to 120 min, depending on the tested species. For *S. maltophilia*, increasing IPM concentrations of 32, 64, 128 mg/L, were used. Negative controls were wells containing bacterial suspensions without IPM.

#### Sample Processing for Imaging

After incubation, each well was cyto-centrifuged on glass slides (Thermo Electron Corporation - Shandon Cytospin 4®) at 800 rpm for 5 min. At this speed, the centrifugation is safe, as no morphological changes of the bacterium are recorded.

All samples underwent the same procedures regarding sample preparation, screening, image acquisition and post-acquisition analysis hereby described (Figure 1). All experiments were performed in triplicate.

#### Imaging Process

Micrographs were recorded using Hitachi's TM4000Plus tabletop SEM. The specificity of this microscope lies in its ease of use for sample visualization and micrograph acquisition compared to older SEMs that require much more expertise. TM4000Plus can ensure the necessary vacuum conditions in <2 min, significantly reducing the images acquisition time compared to other SEMs. At least 10 micrographs were acquired per condition. Accelerating voltages of TM4000Plus was at 15 kV, and the magnification ranged between 2,000 X and 10,000 X. Low magnifications were used for quantification, however high magnification was essential for ultra-structural and morphological investigations. We used the automated imaging function in the TM4000Plus software that enables automated screening and image acquisition.

#### Post-acquisition Analysis Strategy

We looked for morphological modifications of bacteria incubated with or without IPM. Analysis of the bacterial morphology with regards to the shape, density, thickness, brightness signal, formation of blebs, bacterial length, and width were analyzed for all tested bacterial isolates. Measurement of bacterial length and width were performed on the acquired micrographs using Fiji's Image-J software. One hundred bacteria per condition and per isolate were measured by two independent operators on the three sets of data for the experiments carried out in triplicate. The length or width variation between different conditions were considered significant when it was more than 0.2 μm and *p* ≤ *0.001*. Average size ± standard deviations (SD) were used to compare bacteria incubated with or without IPM at the different time points (30, 60, 90, 120 min). Increase or decrease in the brightness signal was also analyzed on Image-J using the greyscale histogram option. We analyzed the contrast of 30 bacteria per condition, extracted the contrast histograms for each bacterium, and calculated the relative SD corresponding to the contrast variation in a single bacterium. Bacterial count was also realized on Image-J using the multi-point tool. We compared the average measurements by performing an ANOVA statistical analysis using ElliStat software. The reported error was set at 95% confidence interval.

### Proof of Concept Validation: Blind Test on Clinical Isolates

Finally, we applied our method blindly to a collection of 58 clinical isolates (Supplementary Table 1). The same strategy of analysis and judgment was carried out to determine the isolates' response toward IPM.

## Results

### Developmental Stage

Identification and susceptibility to IPM of the 13 reference isolates were confirmed by MALDI-TOF/MS and E-test as gold standard. Moreover, we detected different known resistance genes toward carbapenems in the resistant isolates tested, suggesting different resistance mechanisms (Supplementary Table 1).

**Figure 2:**
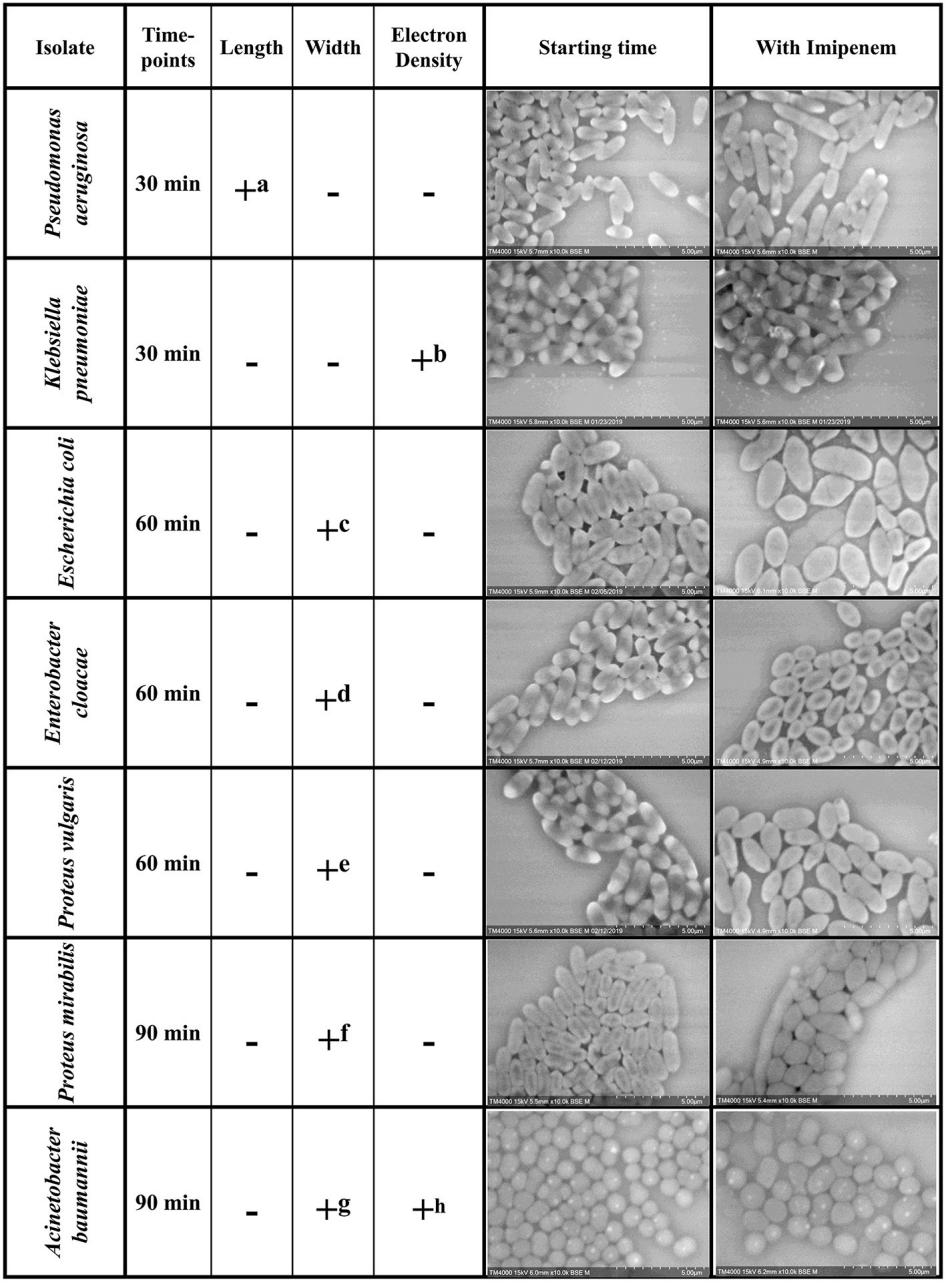
Summary of the morphological modifications associated with susceptibility to IPM for each of the selected species. +: criteria detected; −: criteria not detected **(a)** Increase in *P. aeruginosa* bacterial lengths in the susceptible isolates after 30 min of incubation with IPM. **(b)** Loss of the brightness signal at the extremities in *K. pneumoniae* susceptible isolates after 30 min of incubation with IPM. **(c-f)** Increase in bacterial diameter; emergence of an ovoid shape in susceptible isolates after 60 min of incubation with IPM. **(g)** Inflated *A. baumannii* greatly outnumbers regular sized ones compared to resistant isolates. **(h)** The number of *A. baumannii* showing hyperdense dots decreases in the susceptible isolates and increases in the resistant ones.

We were able to detect discriminating modifications and associated an altered morphology after incubation with IPM to the susceptible isolates, whereas the resistant isolates remained unchanged. The morphological changes observed are regrouped by species in Figure 2. We then extended the incubation time for another 30 min to validate the susceptibility or resistance of the isolate by detecting major structural alterations in the susceptible case only.

#### Bacterial Length Analysis

**Figure 3:**
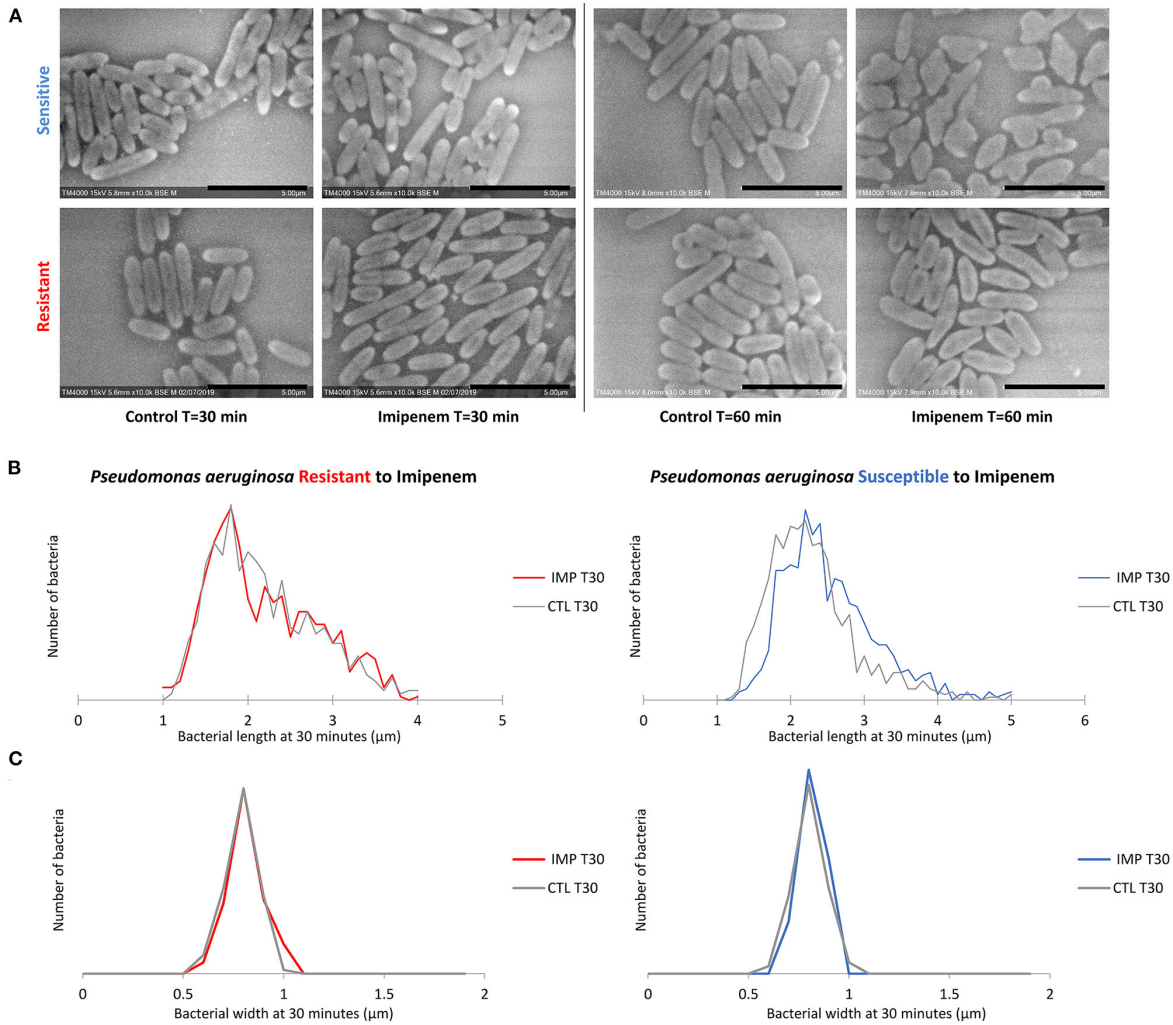
Modifications observed on *P. aeruginosa* incubated with and without IPM for 30 and 60 min. **(A)** Micrographs recorded on TM4000 Plus. Amorphous shapes observed at 60 min in the susceptible isolates confirm the susceptibility of that isolate toward IPM. **(B)** Histograms showing length measurements at 30 min of *P. aeruginosa* for both susceptible and resistant isolates. **(C)** Histograms showing width measurements at 30 min of *P. aeruginosa* for both susceptible and resistant isolates. Scale bars: 5 μm.

Post-acquisition analysis on Image-J showed that the resistant and susceptible strains at the starting point were not statistically different [*p* = *0.9916*]. Also, there was an increase in the bacterial length in susceptible *P. aeruginosa* isolates when incubated with IPM for 30 min at 37°C compared to the control incubated without IPM (susceptible *P. aeruginosa* without and with IPM at T30min: 2.24 ± 0.61 μm; 2.50 ± 0.68 μm, respectively [*p* = *3.032 E-15*]), whereas no significant changes were observed for the resistant isolates in the same conditions [*p* = *0.6442*] (Figures 2, 3A). We followed these morphological modifications to 60 min and observed a clear susceptibility in *P. aeruginosa*, with dominating amorphous shapes that were not measurable on the acquired micrographs, while the resistant strain incubated with IPM were still in shape (Figures 3B,C). For *K. pneumoniae*, length measurements at 30 min were insufficient to discriminate between susceptible and resistant isolates (length of susceptible and resistant *K. pneumoniae* at T0 [*p* = *0.9382*]; susceptible *K. pneumoniae* without and with IPM at T30min [*p* = *0.1115*]; resistant *K. pneumoniae* without and with IPM at T30min [*p* = *0.0091*]). *E. coli, E. cloacae, P. vulgaris, P. mirabilis*, and *S. maltophilia* showed no significant increase in length and no morphological alterations were observed at 30 min.

#### Bacterial Electron Density Analysis

Regarding *K. pneumoniae*, there was a significant loss of the brightness signal at the extremities in the susceptible isolates when incubated with IPM for 30 min at 37°C compared to the control incubated without IPM, whereas it remained bright for the resistant isolates (Figure 2). The greyscale profile of the bacterium validated these results on the 30 min micrographs (Supplementary Figure 1D); with the IPM-susceptible isolate showing a significant decrease in relative SD, validated by a Student *T*-test. We followed the modifications caused by IPM on both *K. pneumoniae* isolates for up to 60 and 90 min, and the effects were clear, as we detected significant morphological changes and bacterial engulfment in the susceptible strain only (Supplementary Figure 1). For *A. baumannii*, we observed hyperdense dots on the bacteria in all isolates. When calculating the ratio: number of dots/ number of bacteria, we noticed the decrease of these structures after 2 h from 78.47 to 51.26% and from 54.46 to 90.83% in the susceptible and the resistant isolates, respectively, suggesting a morphological modification of *A. baumannii* after contact with imipenem for 120 min (Supplementary Figure 2D).

#### Bacterial Width Analysis

**Figure 4:**
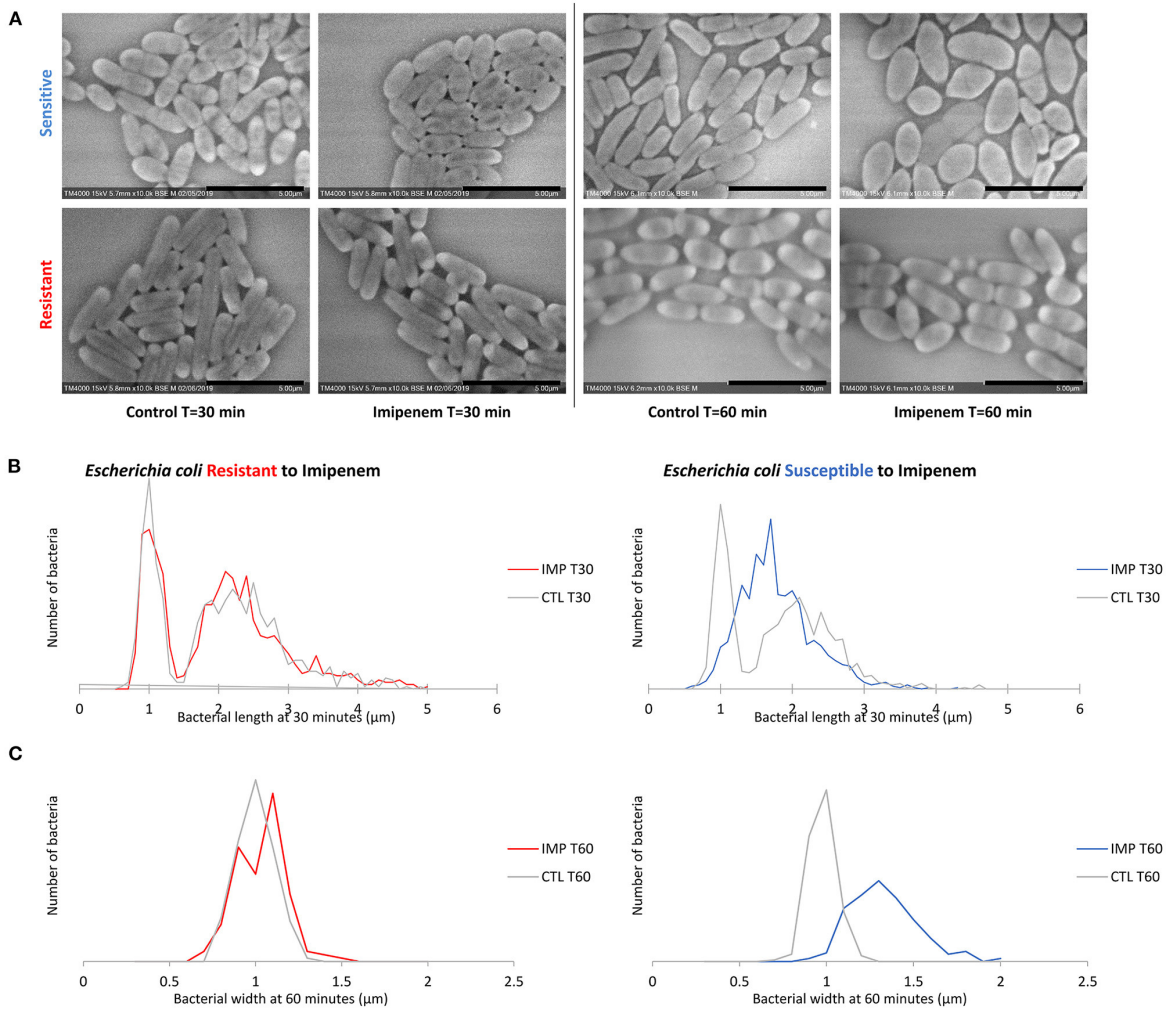
Modifications observed on *E. coli* incubated with and without IPM for 30 and 60 min. **(A)** Micrographs recorded on TM4000 Plus. Bacterial inflation and damage observed at 60 min in the susceptible isolates confirms the susceptibility of that isolate toward IPM. **(B)** Histograms showing length measurements at 30 min of *E. coli* for both susceptible and resistant isolates. **(C)** Histograms showing width measurements at 60 min of *E. coli* for both susceptible and resistant isolates. Scale bars: 5 μm.

**Figure 5:**
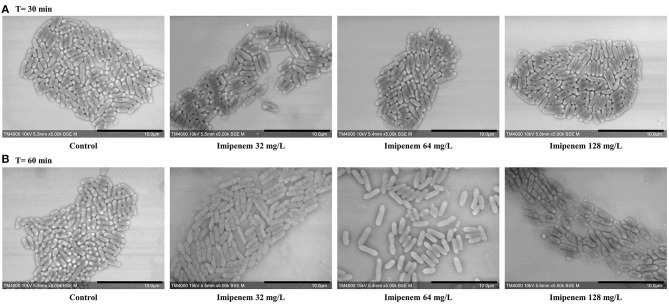
Modifications observed on *S. maltophilia* incubated without IPM and with IPM. **(A)** After 30 min. **(B)** After 60 min. No significant morphological modifications were observed after incubation with increasing IPM concentrations for up to 60 min. Micrographs recorded on TM4000 Plus. Scale bars: 10 μm.

When evaluating bacterial width, we noticed an increase in width in susceptible *P. aeruginosa* after incubation with IPM for 30 min, although the length measurements alone were sufficient to discriminate between susceptible and resistant isolates. For *E. coli* and *E. cloacae*, the bacterial width showed a major increase in the diameter of the susceptible isolates which was detectable after 60 min of incubation (susceptible *E. coli* without and with IPM at T60min: 0.92 ± 0.07 μm; 1.28 ± 0.19 μm respectively [*p* = *1.242E-103*]; susceptible *E. cloacae* without and with IPM at T60min: 0.90 ± 0.11 μm; 1.24 ± 0.19 μm respectively [*p* = *1.814E-88*]) while the resistant isolates maintained a stable width when incubated with IPM for up to 90 min (resistant *E. coli* [*p* = *0.0139*]; resistant *E. cloacae* [*p* = *0.8078*]). These modifications allowed us to differentiate susceptible from resistant isolates after 60 min of incubation (Figure 4A and Supplementary Figure 3A). On the acquired micrographs, these modifications are explained by the emergence of an ovoid shape of the susceptible bacterium when incubated with IPM for 60 min, and amorphous shapes at 90 min, whereas the resistant isolates showed no response to IPM (Figures 4B,C and Supplementary Figure 3B). The micrographs of the susceptible *P. vulgaris* isolates showed large numbers of bacteria expressing an ovoid shape after 60 min of incubation with IPM. An increase in bacterial width compared to the control which was incubated without an antibiotic was detected at 60 min (susceptible *P. vulgaris* without and with IPM at T60min: 0.83 ± 0.09 μm; 1.05 ± 0.13 μm, respectively [*p* = *1.107E-15*]) (Supplementary Figure 4A). Resistant isolates seemed to undergo the same effect but at a slower rate: when incubated with IPM for 90 min at 37°C, the ovoid shape became dominant (Supplementary Figure 4B). The micrographs of the susceptible *P. mirabilis* isolates showed that even though ovoid shapes appeared after 60 min of incubation with antibiotic (Supplementary Figure 5A), the increase in bacterial width compared to the control was detected only after 90 min (susceptible *P. mirabilis* without and with IPM at T90min: 0.80 ± 0.09 μm; 1.07 ± 0.18 μm, respectively [*p* = *9.03E-29*]) (Supplementary Figure 5B). The susceptible isolates of *A. baumannii* showed an increased diameter at 90 min (Supplementary Figure 2A) that could not be detected in the resistant *A. baumannii*, enabling us to separate susceptible and resistant bacteria after 90 min of incubation with IPM (Supplementary Figure 2B). The last tested species in this study was *S. maltophilia* that showed no morphological modification when incubated for up to 60 min with increasing IPM concentrations, and when post-acquisition analysis was applied for length, width, and brightness; no significant differences compared to the control were detected (Figure 5).

### Proof of Concept

When analyzed blindly, all 58 tested clinical isolates were correctly classified as susceptible or resistant toward IPM when comparing our results to the routinely used AST methods. The whole process from bacterial culture to the results yield took <2 h to evaluate the microbe's response toward IPM going from the liquid culture until results readout.

## Discussion

In this work, we were able to distinguish between resistant and susceptible isolates of clinically relevant Gram-negative bacteria (Kadri et al., 2018; Strich and Kadri, 2019). We demonstrated that after 30 to 90 min of contact with IPM morphological alterations were detected and associated to a susceptible profile, whereas resistant isolates were not affected, retaining the same shape even when in contact with antibiotics. IPM affects the bacterial inner membrane's penicillin binding protein, altering cell wall synthesis, explaining the changes in bacterial ultra-structure (Williams et al., 1986). Certain parameters were proposed before this study, such as the increase in length, frequently observed with β-lactams resulting in filament formation and emergence of spheroplasts with longer incubation times, or an increase in diameter, followed by the bacterium bursting (Choi et al., 2017). Moreover, other studies recently evaluated the phenotypic response of different bacterial species using phase contrast microscopy. Diverse abnormal morphologies were associated to *K. pneumoniae* after incubation with a panel of antibiotics, notably analyzing the length to width ratio and cell roundness among other parameters (Sridhar et al., 2021). Also, bulging was noticed in *E. coli* isolates after a short incubation with cefsulodin, another β-lactam antibiotic, and was observed right before the cell lysis (Zahir et al., 2019).

One of the most surprising findings in our study was the disappearance of bipolar brightness, which occurred simultaneously with bacterial multiplication, and regressed when antibiotics were active on *K. pneumoniae*. This newly developed AST was proven to be reproducible when performed in triplicate. These preliminary results should be confirmed with other carbapenem-resistant isolates presenting various resistance mechanisms or heterogeneous resistance (Andersson et al., 2019). However, further analysis is needed for this strategy to allow the determination of the MIC breakpoint for the tested strain, and to create a panel able to determine intermediately-resistant isolates. Another drawback is that the incubation time before detection of visible morphological modifications is species dependent and can be extended with slow-growth bacteria. This work proves that SEM could allow identifying changes in bacterial morphology at a very early growth stage, as opposed to other methods where longer incubation times are needed to detect the resistant bacteria. The objective of this study was to demonstrate as a proof of concept the ability of this rapid AST using SEM to identify morphological changes associated with susceptibility to IPM in clinically relevant Gram-negative bacteria (Strich and Kadri, 2019) within a short time. A larger number of frequently found microbes and their response to other molecules need to be further analyzed before applying this method directly on positive blood cultures, allowing faster antimicrobial susceptibility diagnosis therefore limiting the broad-spectrum antibiotic use until the availability of the test results.

This method allowed to improve the AST of Gram-negative bacilli toward IPM, where the whole process from sample preparation to results yield takes 1 to 2 h depending on the specie, whereas for the routinely used techniques, results can take up to 48 h before judgment. Novel technologies available on the market also reduced the AST time-to-result, such as Quantamatrix direct rapid AST yielding results after 6 h (Wang et al., 2017), Accelerate Pheno-Systems generating AST results within 7 h (Charnot-Katsikas et al., 2018; De Angelis et al., 2019), and Alifax Alfred60 AST with a time-to-result of 5 h (Boland et al., 2019; Van den Poel et al., 2020). However, costs are very high to be applied globally, especially in under-developed countries. Given the low cost of this type of device and the very high handling speed, this work opens up a new era which could revolutionize microbiological diagnosis, raising one the most important emerging problems in the management of infectious diseases.

This strategy is a potential candidate to be developed into a fully automated system for high throughput antibiotic susceptibility testing applied in clinical microbiology, capable of responding to urgent diagnostic questions.

## Data Availability Statement

The original contributions presented in the study are included in the article/Supplementary Material, further inquiries can be directed to the corresponding author/s.

## Ethics Statement

The research ethics committee of the Institut Hospitalo-Universitaire (IHU) Méditerranée Infection was consulted for the experimental work undertaken. No ethical approval or permits were required for conducting the research reported in this manuscript according to the laws and regulations.

## Author Contributions

DR and JB conceived and designed the experiments. GH, AF, JB, and AH analyzed the data. GH and JB wrote the paper. SB, JB, YO, J-MR, and DR revised the paper. All authors contributed materials/analysis tools.

## Conflict of Interest

DR was a consultant in microbiology for the Hitachi High-Tech Corporation. TT and YO were employed by the company Hitachi High-Tech Corporation. AH was employed by the company Hitachi, Ltd. Personal fees of GH, AF and JB are paid with a grant from the company Hitachi High-Tech Corporation. The remaining authors declare that the research was conducted in the absence of any commercial or financial relationships that could be construed as a potential conflict of interest. A patent application relating to this research work is pending. IHU and Hitachi High-Tech have the right to obtain a patent.
