# Supplementary material for: Rapid Detection of Imipenem Resistance in Gram-Negative Bacteria Using Tabletop Scanning Electron Microscopy: A Preliminary Evaluation
Source: Front Microbiol. 2021 Jun 16;12:658322. doi: 10.3389/fmicb.2021.658322 (PMC8245003; doi:10.3389/fmicb.2021.658322)
Supplement: Supplementary file 1 [file Data_Sheet_1.pdf]

SUPPLEMENTARY DATA

(A) Reference isolates

Species	CSUR reference number	Imipenem resistance profile	EUCAST interpretation	E-test	Resistance gene
Pseudomonas aeruginosa	CSURP9558	Susceptible	4mg/L	1mg/L	N/A
	CSURP9559	Resistant		>32mg/L	OprD truncated
Klebsiella pneumoniae	CSURP9552	Susceptible	2mg/L	0.19mg/L	N/A
	CSURP9553	Resistant		8mg/L	blaVIM-1
Escherichia coli	CSURP9547	Susceptible	0.5mg/L	0.25mg/L	N/A
	CSURP9546	Resistant		1.5mg/L	blaNDM-1
Enterobacter cloacae	CSURP9549	Susceptible	1mg/L	0.25mg/L	N/A
	CSURP9548	Resistant		2mg/L	blaNDM-1
Proteus vulgaris	CSURP9556	Susceptible	4mg/L	2mg/L	N/A
	CSURP9557	Resistant		>32mg/L	unknown
Acinetobacter baumannii	CSURP9541	Susceptible	1mg/L	0.125mg/L	N/A
	CSURP9540	Resistant		>32mg/L	blaOxa-24
Stenotrophomonas maltophilia	CSURP5256	Resistant	N/A	>32mg/L	Naturally resistant

(B) Clinical isolates

Species	Isolate nature	Imipenem resistance profile	EUCAST interpretation	E-test	Resistance gene
Pseudomonas aeruginosa	CSURQ5591	Susceptible	4mg/L	1.5mg/L	N/A
	CSURQ5592	Susceptible		1.5mg/L	N/A
	CSURQ5593	Susceptible		0.38 mg/L	N/A
	CSURQ5594	Susceptible		1.5mg/L	N/A
	CSURQ5330	Susceptible		1.5 mg/L	N/A
	CSURQ5595	Resistant		>32mg/L	Unknown
	CSURQ5596	Resistant		>32mg/L	blaVIM-1
Klebsiella pneumoniae	CSURQ5561	Susceptible	2mg/L	0.75mg/L	N/A
	CSURQ5565	Susceptible		0.25mg/L	N/A
	CSURQ5566	Susceptible		0.5mg/L	N/A
	CSURP9554	Susceptible		0.25 mg/L	N/A
	CSURQ5568	Susceptible		0.19mg/L	N/A
	CSURQ5569	Susceptible		0.125mg/L	N/A
	CSURQ5562	Resistant		2 mg/L	blaOxa-48
	CSURQ5563	Resistant		4mg/L	blaNDM-1
	CSURQ5564	Resistant		1.5 mg/L	blaVIM-1
	CSURQ5567	Resistant		>32mg/L	blaNDM-1
	CSURQ5570	Resistant		1.5 mg/L	blaOxa-48
Escherichia coli	CSURP1767	Susceptible	0.5mg/L	0.19 mg/L	N/A
	CSURQ5583	Susceptible		0.064mg/L	N/A

Escherichia coli	CSURQ5584	Susceptible	0.5mg/L	0.125mg/L	N/A
	CSURQ5585	Susceptible		0.125mg/L	N/A
	CSURQ5586	Susceptible		0.19 mg/L	N/A
	CSURQ5587	Susceptible		0.125 mg/L	N/A
	CSURQ5588	Susceptible		0.19 mg/L	N/A
	CSURQ5589	Susceptible		0.125mg/L	N/A
	CSURQ5590	Susceptible		0.19mg/L	N/A
	CSURQ5581	Resistant		0.75mg/L	blaOxa-48
	CSURQ5582	Resistant		>32mg/L	blaNDM-1
	CSURP5238	Resistant		0.75mg/L	blaOxa-48
	CSURP1872	Resistant		32mg/L	blaNDM-1
	CSURP1954	Resistant		32mg/L	blaNDM-1
Enterobacter cloacae	CSURQ5598	Susceptible	1mg/L	0.25 mg/L	N/A
	CSURQ5599	Susceptible		0.25 mg/L	N/A
	CSURQ5601	Susceptible		0.25 mg/L	N/A
	CSURQ5602	Susceptible		0.38 mg/L	N/A
	CSURQ5603	Susceptible		0.25mg/L	N/A
	CSURQ5604	Susceptible		0.25 mg/L	N/A
	CSURQ5606	Susceptible		0.25 mg/L	N/A
	CSURQ1030	Resistant		1.5 mg/L	blaOxa-48
	CSURQ5600	Resistant		12 mg/L	unknown
	CSURQ5605	Resistant		1.5 mg/L	blaOxa-48
	CSURP1944	Resistant		1 mg/L	blaOxa-48
Proteus vulgaris	CSURQ5597	Susceptible	4mg/L	0.25 mg/L	N/A
Proteus mirabilis	CSURP2049	Susceptible	4mg/L	0.38 mg/L	N/A
	CSURP5544	Susceptible		0.5 mg/L	N/A
	CSURP8539	Resistant		>32mg/L	blaNDM-1
	CSURP8541	Resistant		>32mg/L	blaNDM-1
Acinetobacter baumannii	CSURQ5572	Susceptible	1mg/L	0.25mg/L	N/A
	CSURQ5577	Susceptible		0.25mg/L	N/A
	CSURQ5578	Susceptible		0.125mg/L	N/A
	CSURQ5579	Susceptible		0.094mg/L	N/A
	CSURQ5580	Susceptible		0.25 mg/L	N/A
	CSURQ5571	Resistant		1.5mg/L	blaOxa-23
	CSURQ5573	Resistant		>32mg/L	blaOxa-23
	CSURQ5574	Resistant		6mg/L	blaNDM-1
	CSURQ5575	Resistant		>32mg/L	blaOxa-23
	CSURQ5576	Resistant		>32mg/L	blaOxa-23

Table S1. Tested isolates and their resistance profile to imipenem. The liquid Minimal Inhibitory Concentrations (MIC) recovered from EUCAST. The E-test results and the resistance gene present in each isolate were validated by RT-PCR. **(A)**. Reference isolates; **(B)**. Clinical isolates.

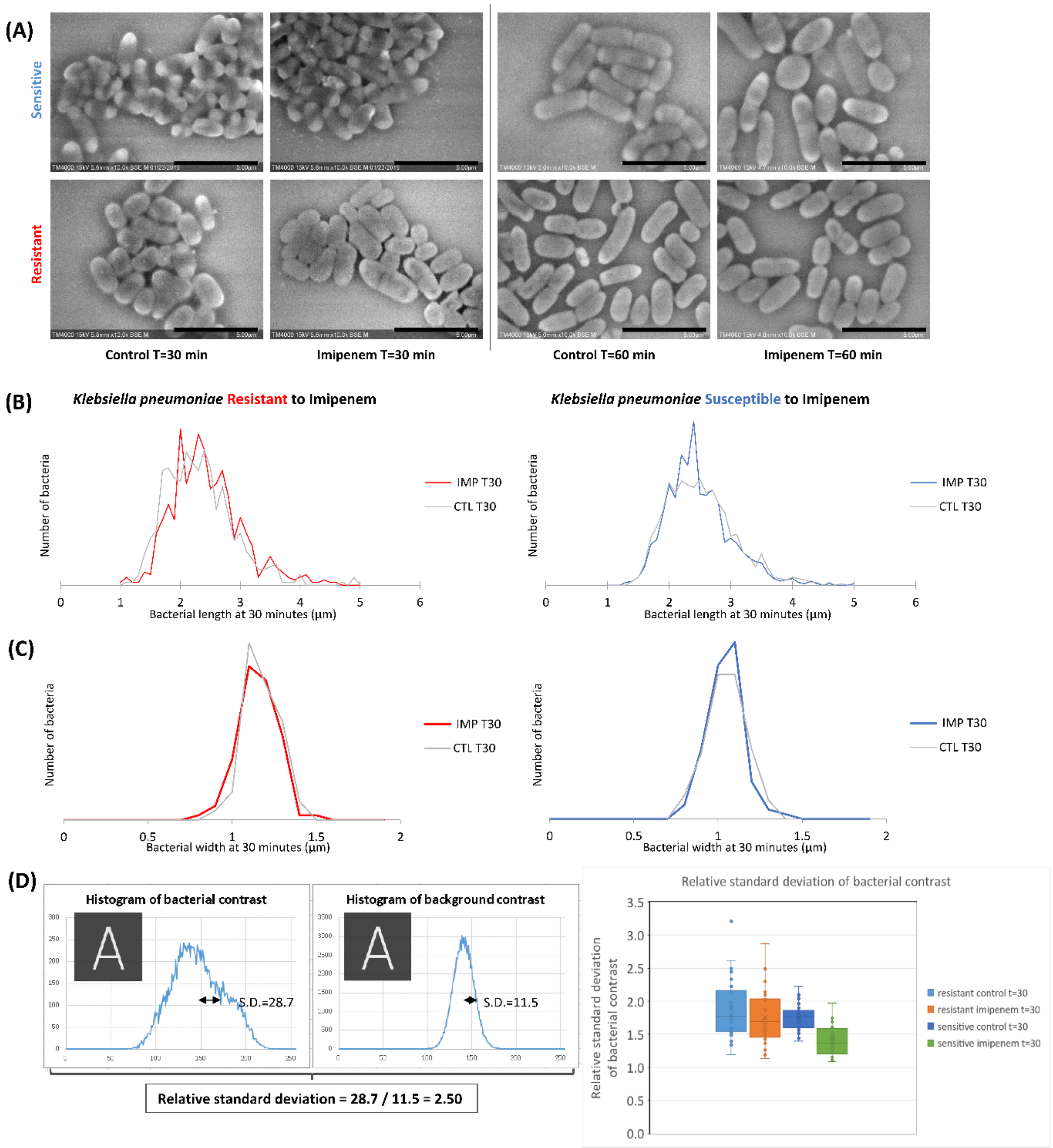

Figure S1. Modifications observed on *K. pneumoniae* incubated with and without IPM for 30 and 60 minutes. (A). TM4000Plus micrographs. Bacterial damage: engulfment formation observed at 60 minutes in the susceptible isolates confirms the susceptibility of that isolate towards IPM. **(B)**. Histograms showing length measurements at 30 minutes of *K. pneumoniae* for both susceptible and resistant isolates. **(C)**. Histograms showing width measurements at 30 minutes of *K. pneumoniae* for both susceptible and resistant isolates. **(D)**. Histograms showing brightness measurements for a single bacterium and for the background at 30 minutes of *K. pneumoniae* and the relative SD of the bacterial contrast when compared to the background for both susceptible and resistant isolates. Scale bars: 5µm.

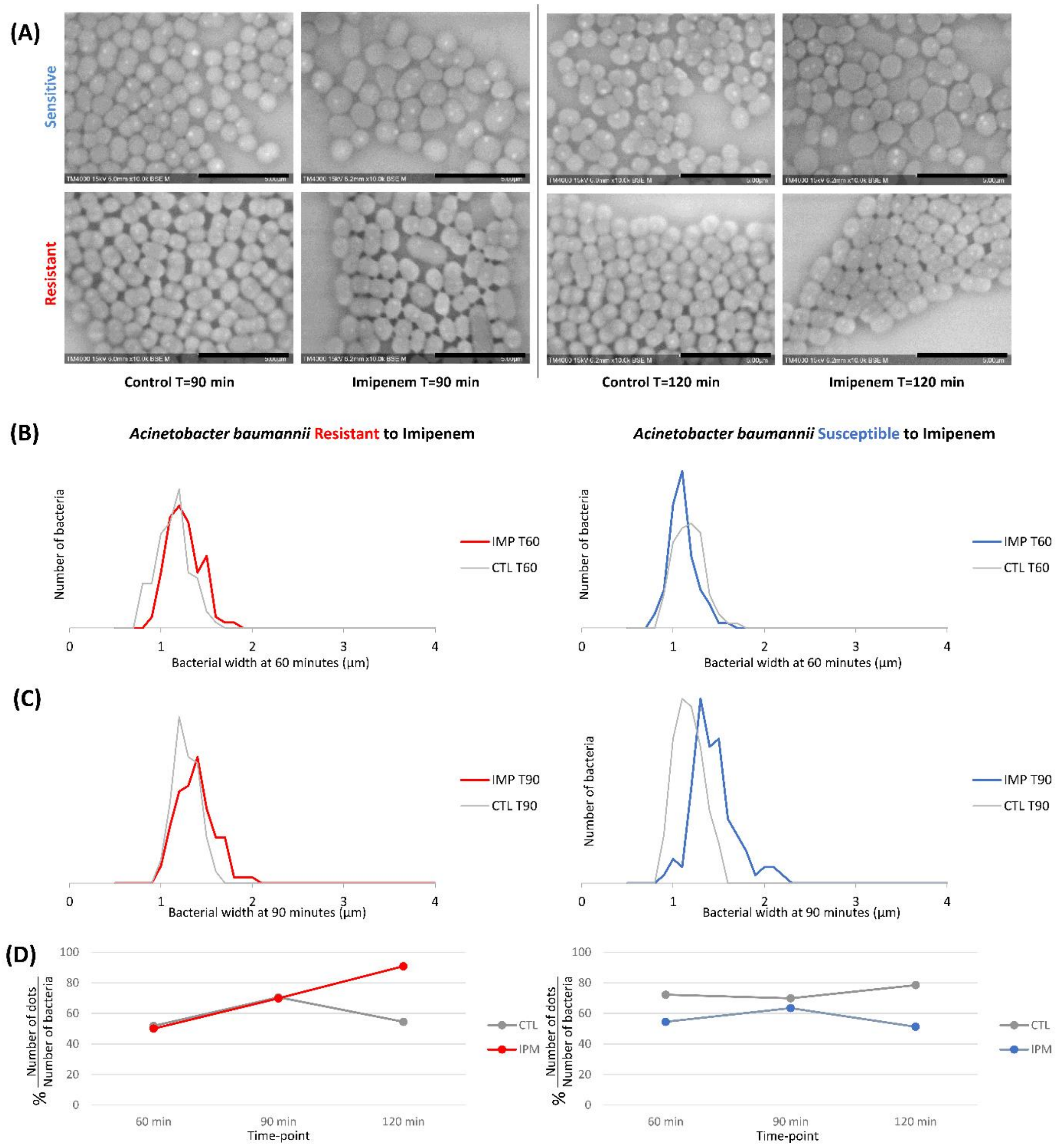

Figure S2. Modifications observed on *Acinetobacter baumannii* incubated with and without Imipenem for 90 and 120 minutes. (A). TM4000Plus micrographs. Inflated bacteria outnumber greatly the regular sized ones in the susceptible isolates confirming the susceptibility of that isolate towards Imipenem. **(B). (C).** Histograms showing the width measurements at 60 and 90 minutes of *A. baumannii* for both susceptible and resistant isolates. **(D).** Total bacteria and hyperdense dots count at 60, 90 and 120 minutes for both susceptible and resistant isolates. Scale bars: 5µm.

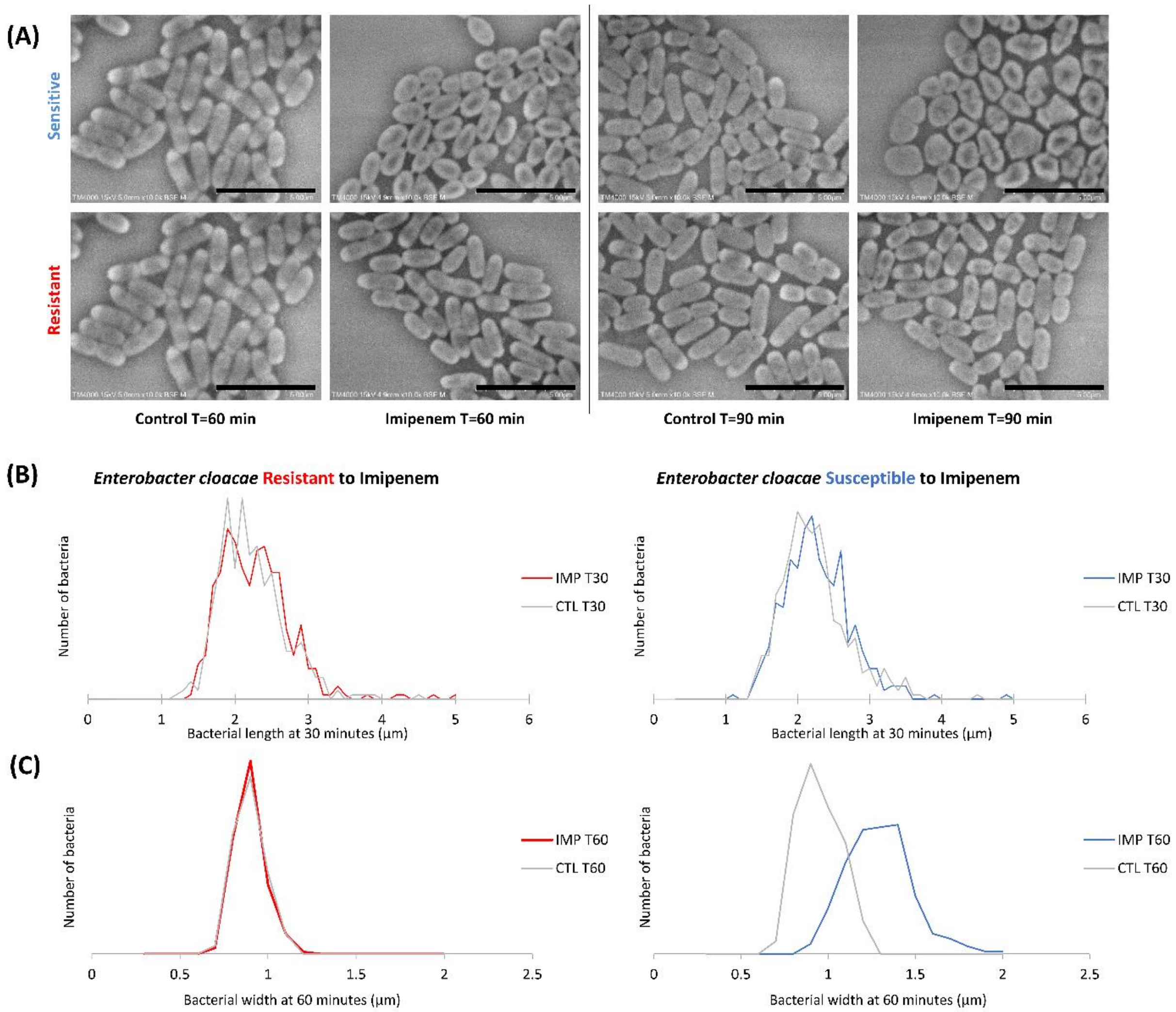

Figure S3. Modifications observed on *Enterobacter cloacae* incubated with and without imipenem for 60 and 90 minutes. (A). TM4000Plus micrographs. Amorphous shapes observed at 60 minutes in the susceptible isolates confirm the susceptibility of that isolate towards imipenem. **(B)**. Histograms showing length measurements at 30 minutes of *E. cloacae* for both susceptible and resistant isolates. (**C)**. Histograms showing width measurements at 60 minutes of *E. cloacae* for both susceptible and resistant isolates. Scale bars: 5µm.

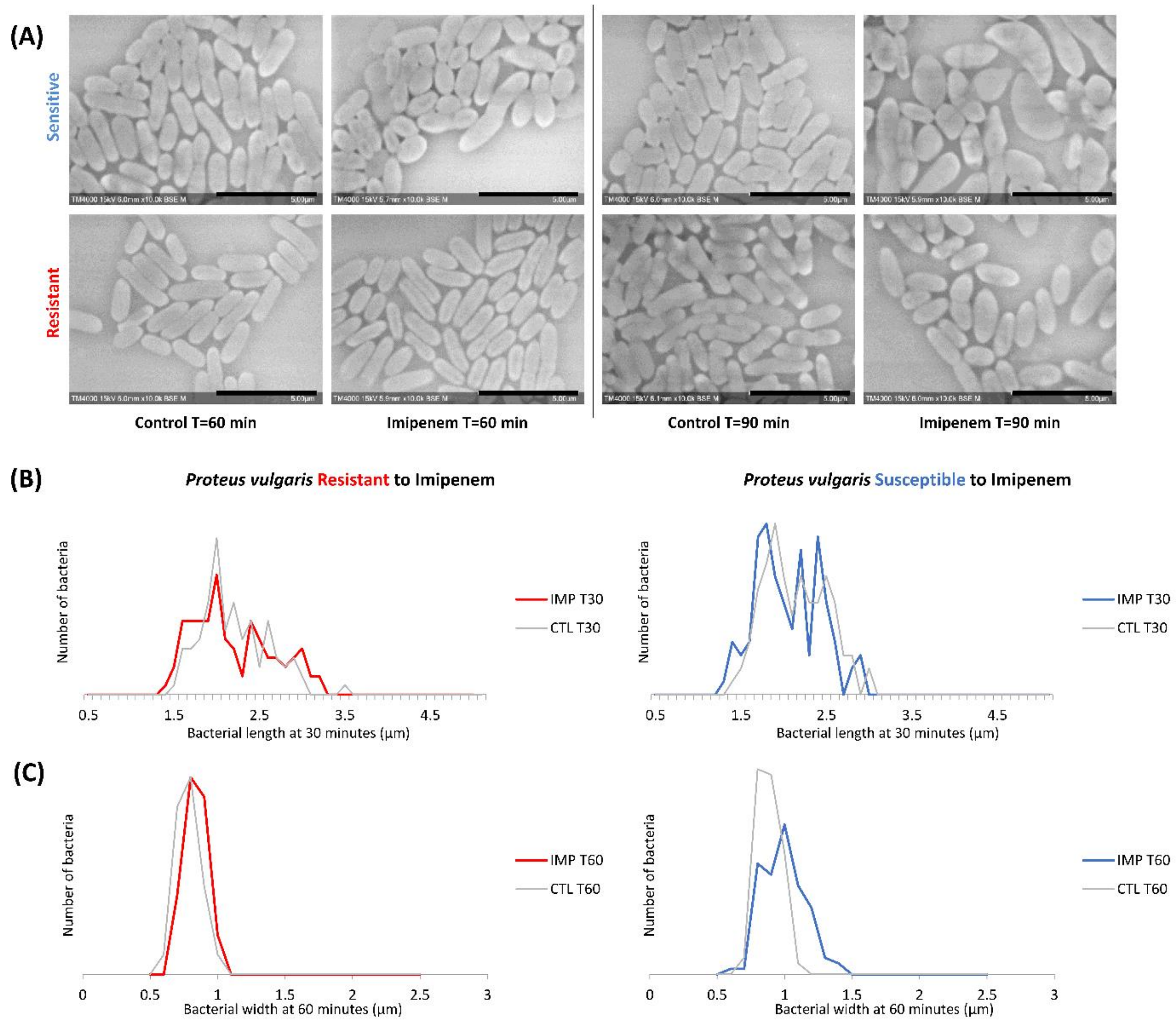

Figure S4. Modifications observed on *Proteus vulgaris* incubated with and without imipenem for 60 and 90 minutes. (A). TM4000Plus micrographs. Bacterial inflation and damage observed at 60 minutes in the susceptible isolates confirms the susceptibility of that isolate towards imipenem. **(B).** Histograms showing length measurements at 30 minutes of *P. vulgaris* for both susceptible and resistant isolates. **(C).** Histograms showing width measurements at 60 minutes of *P. vulgaris* for both susceptible and resistant isolates. Scale bars: 5µm.

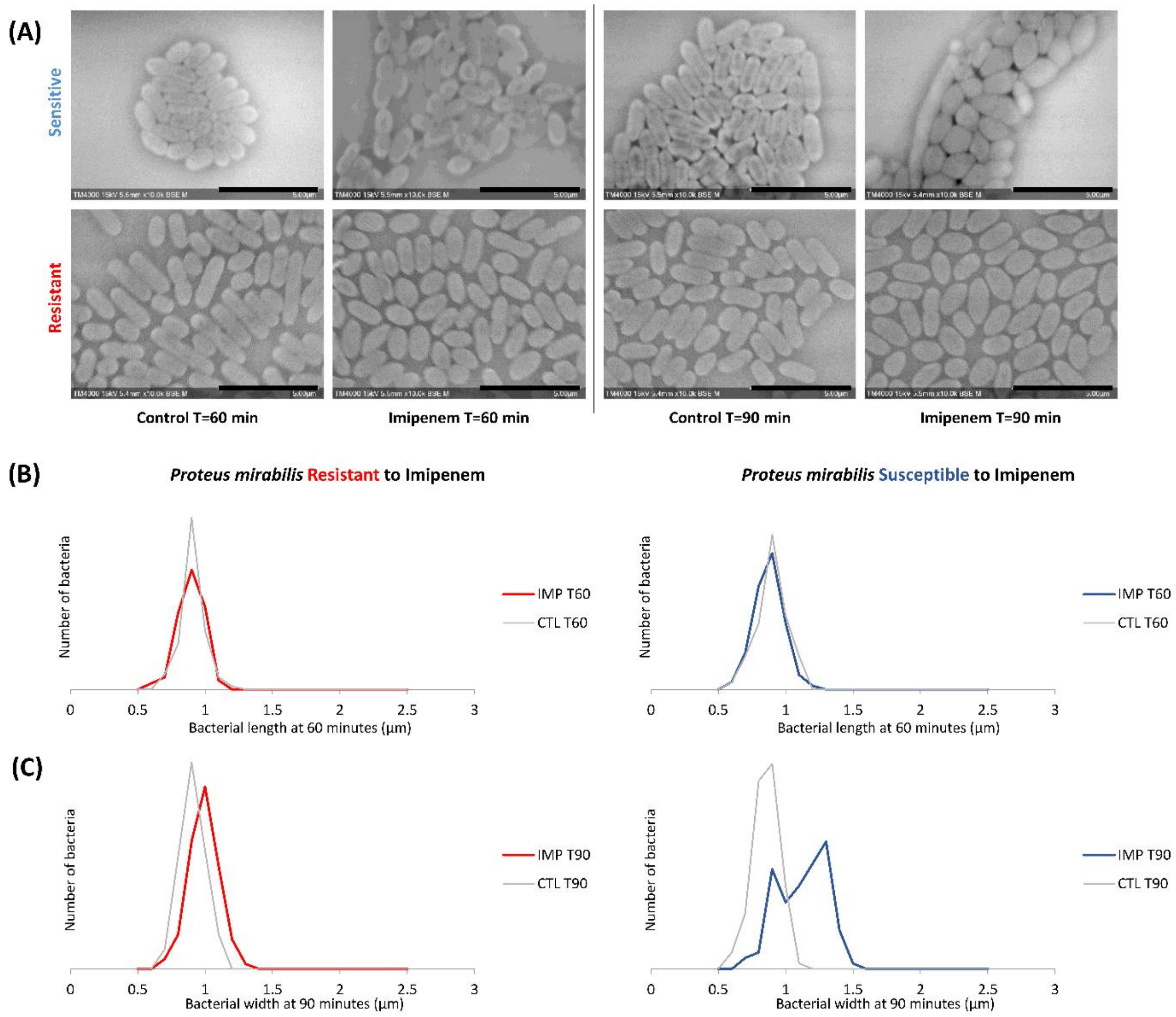

Figure S5. Modifications observed on *Proteus mirabilis* incubated with and without imipenem for 60 and 90 minutes. (A). TM4000Plus micrographs. Bacterial inflation and damage observed at 60 minutes in the susceptible isolates confirms the susceptibility of that isolate towards imipenem. **(B).** Histograms showing length measurements at 60 minutes of *P. mirabilis* for both susceptible and resistant isolates. **(C).** Histograms showing width measurements at 90 minutes of *P. mirabilis* for both susceptible and resistant isolates. Scale bars: 5µm.
